# The alterations of retinal vasculature detected on optical coherence tomography angiography associated with chronic obstructive pulmonary disease

**DOI:** 10.1111/crj.13478

**Published:** 2022-02-11

**Authors:** Murat Serkan Songur, Yavuz Selim İntepe, Seray Aslan Bayhan, Hasan Ali Bayhan, Bülent Çiftçi, Mehmet Çıtırık

**Affiliations:** ^1^ Department of Ophthalmology, Faculty of Medicine Yozgat Bozok University Yozgat Turkey; ^2^ Department of Chest Disease, Faculty of Medicine Yozgat Bozok University Yozgat Turkey; ^3^ Ankara Ulucanlar Eye Training and Research Hospital University of Health Sciences Ankara Turkey

**Keywords:** chronic obstructive pulmonary disease, deep capillary plexus, foveal avascular zone, optical coherence tomography angiography, vascular density

## Abstract

**Objectives:**

This study aimed to evaluate the retinal vasculature of the macula and optic disc in patients with chronic obstructive pulmonary disease (COPD) by optical coherence tomography angiography (OCTA).

**Methods:**

The right eyes of 70 COPD patients and 71 healthy individuals were evaluated. These patients had moderate airflow limitation and mean PO_2_ of 60 mmHg, and their average age was less than 60 years. Superficial and deep capillary plexus vascular densities, foveal avascular zone (FAZ) width, and optic disc parameters were measured with OCTA. In addition, the correlation between the PO_2_ level in COPD patients and superficial, deep, and peripapillary vascular densities and FAZ was examined in the study.

**Results:**

The COPD group had a significant decrease in the vascular density in the superficial (fovea [*p* = 0.019]; parafovea [*p* = 0.013]; and perifovea [*p* = 0.001]) and deep capillary plexus (fovea [*p* = 0.028]; parafovea [*p* = 0.005]; and perifovea [*p* = 0.002]). Also, the enlargement of the FAZ (*p* = 0.002) and a decrease in the peripapillary vascular density (*p* = 0.006) were observed in the COPD group. There was a positive correlation between PO_2_ level and superficial, deep, and peripapillary vascular densities in COPD patients and a negative correlation with FAZ (*r* = 0.559–0.900).

**Conclusion:**

Hypercapnia, respiratory acidosis, and chronic hypoxia associated with COPD may affect the macula and optic nerve, resulting in a serious decrease in vascular density, and OCTA can be a very important tool in the follow‐up and treatment of these patients.

## Graphical abstract

To evaluate the retinal vasculature of the macula and optic disc in patients with chronic obstructive pulmonary disease (COPD) by optical coherence tomography angiography (OCTA).The right eyes of 70 COPD patients and 71 healthy individuals were evaluated. Superficial and deep capillary plexus vascular densities, foveal avascular zone (FAZ) width, and optic disc parameters were measured with OCTA. The COPD group had a significant decrease in the vascular density in the superficial (fovea (*p* = 0.019); parafovea (*p* = 0.013); perifovea (*p* = 0.001)) and deep capillary plexus (fovea (*p* = 0.028); parafovea (*p* = 0.005); perifovea (*p* = 0.002)). Also, the enlargement of the FAZ (*p* = 0.002), and a decrease in the peripapillary vascular density (*p* = 0.006), were observed in the chronic obstructive pulmonary disease group. There was a positive correlation between pO_2_ level and superficial, deep and peripapillary vascular density in COPD patients, and a negative correlation with FAZ (*r* = 0.559‐0,900). OCTA can be a very important tool in the follow‐up and treatment of these patients.

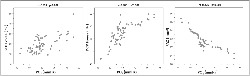

## 1 INTRODUCTION

Chronic obstructive pulmonary disease (COPD) is a progressive disease that begins with the respiratory tract's abnormal inflammatory response to gases and some particles.^1^ This inflammation results in gradual airflow limitation, leading to alveolar hypoxia and subsequently hypoxemia.^2^ In 2006, the Global Initiative for Chronic Obstructive Lung Disease (GOLD) defined COPD as a systemic disease and stated that extrapulmonary effects and additional diseases affect its severity.^3^ The prevalence of COPD in individuals over the age of 40 years is around 9–10%, and it is more common in men.^4^

The diagnosis of COPD is made if the ratio of forced expiratory volume (FEV1) in the first second to forced vital capacity (FVC) is less than 70% in spirometry.^5^ Weight loss and malnutrition are the two most common systemic effects in COPD. However, sleep diseases, diabetes, lower and upper respiratory tract infections, obstructive sleep apnea syndrome, depression, myocardial infarction, and lung cancer are also frequently seen in COPD.^3^

Optical coherence tomography angiography (OCTA) is a noninvasive clinical tool that can capture retinal capillary microcirculation.^6^ It is an imaging method that provides detailed visualization of the retinal vascular network by obtaining and processing the motion contrast of erythrocytes in the vessel with sequential optical coherence tomography (OCT) scans of a certain retinal area. In this angiography method, unlike fundus fluorescent angiography (FFA), no intravenous contrast material is used, and therefore, the procedure is not invasive.^7^ In this study, we tried to investigate the status of the microvascular structure of the retina and peripapillary region and the effect of COPD on these structures by using OCTA.

## 2 MATERIALS AND METHODS

We conducted this study in our hospital lung diseases and ophthalmology outpatient clinics between March 2020 and March 2021. Before starting the study, we obtained informed consent from all patients, and approval was obtained from the ethics committee of our hospital.

All precautions have been taken due to the COVID‐19 pandemic. All COPD and control patients underwent respiratory function tests with filtered spirometry (Carefusion Encore 22D; Sensor Medics, USA) at the pulmonary function test laboratory. The FEV1, FVC, and FEV1/FVC ratios were recorded. All COPD patients were stable without exacerbation in the last 3 months. We used a Siemens RAPIDLab 1265 blood gas analyzer (Siemens, Medfield, MA, USA) to analyze arterial blood gas. The PO_2_, pH, PCO_2_, HCO_3_, and Sat O_2_ values were entered. We obtained arterial blood gases without oxygen support and after 20 min of rest.

In our study, we selected the control group from individuals who did not have systemic disease, had no history of trauma in the eye, had no eye pathology, and could cooperate with OCTA.

A complete ophthalmologic evaluation including intraocular pressure (IOP) measurement, best corrected visual acuity, slit lamp biomicroscopy, gonioscopy with three‐mirror contact lenses, and fundoscopy was performed in all participants. Patients with glaucoma, hypertensive or diabetic retinopathy, epiretinal membrane, corneal opacity, high hypermetropia or myopia (>6D), corneal disease, history of ocular trauma, and surgery and the ones who could not cooperate during OCTA imaging were excluded. In this study, patients with diabetes, patients with dementia, and people with cardiovascular system diseases such as hypertension were excluded from the study.

Foveal avascular zone (FAZ) width, superficial (SCP) and deep capillary plexus (DCP) vascular density (VD), and optic disc and peripapillary VD measurements were performed with the RTVue OCTA system (RTVue XR Avanti System; Optovue Inc, Fremont, CA, USA). Only images with a quality level of ≥7 in OCTA scans were included in the study.

In examinations with OCTA, the SCP and DCP of the retina were evaluated as 6 × 6 mm angioretina. The VDs of the fovea, parafovea, and perifovea were examined in the SCP and DCP. Moreover, we also evaluated the width of the FAZ, which is the region of the fovea that has no vascularization, and the capillary vessel density covering 300 μm around the fovea (FD‐300).

Peripapillary VD was also measured as 4.5 × 4.5 mm with OCTA. Inside disc representing 2 mm center of the optic disc along with eight quadrants was also measured.

We performed statistical analysis using the SPSS® 22.0 package program. Descriptive statistics were made. We used the chi‐squared test to compare categorical variables. Shapiro–Wilk test was used to evaluate the distribution of normality. We used Mann–Whitney *U* test for comparison of non‐normally distributed data and Student's *t* test for binary comparison of normally distributed data. In the correlation analysis, the distribution of the parameters was evaluated by applying Kolmogorov–Smirnov and Shapiro–Wilk tests. Pearson correlation analysis was performed for the parameters that showed normal distribution, and Spearman correlation analysis was performed for those that did not show normal distribution.

## 3 RESULTS

In this study, we evaluated the right eyes of 70 patients diagnosed with COPD and 71 healthy controls; 15 (21.5%) patients were smokers, 43 (61.4%) were ex‐smokers, and 12 (17.1%) were nonsmokers in COPD group. The control group was chosen from nonsmokers. COPD patients in stable period were included in the study. There was no difference in C‐reactive protein (CRP) values between the two groups. Table 1 shows the arterial blood gas values, spirometry values of the participants, CRP value, smoking status, and the sociodemographic characteristics of the patients.

**TABLE 1:** Sociodemographic results, spirometry data, and arterial blood gas analysis of the groups

	Group 1 (*n* = 70)	Group 2 (*n* = 71)	*p* value
Sociodemographic results			
Female	33 (47.1%)	35 (49.2%)	0.555
Male	37 (52.9%)	36 (50.8%)	
Age	59.78 ± 10.63	57.26 ± 12.61	0.202
BMI	29.23 ± 5.49	30.48 ± 5.33	0.281
Smoking (pocket/year)	19.21 ± 12.23	—	
CRP	1.46 ± 0.92	1.65 ± 1.07	0.249
Spirometry data			
FEV1 (%)	60.87 ± 11.25	106.46 ± 21.36	<0.001
FEV1 (L)	1.54 ± 0.64	2.84 ± 0.91	<0.001
FVC (L)	2.58 ± 0.98	3.55 ± 1.06	<0.001
FVC (%)	81.74 ± 21.36	109.77 ± 21.14	<0.001
FEV1/FVC (%)	58.77 ± 7.15	79.01 ± 4.88	<0.001
Arterial blood gas analysis			
PO_2_ (mmHg)	60.29 ± 9.64	75.94 ± 9.69	<0.001
Sat O_2_ (%)	88.13 ± 4.02	94.24 ± 1.87	<0.001
PCO_2_ (mmHg)	43.65 ± 7.18	38.43 ± 3.38	<0.001
HCO_3_ (mmol/L)	26.55 ± 3.51	22.94 ± 2.70	<0.001
pH	7.37 ± 0.04	7.41 ± 0.03	<0.001

*Note*: Continuous data are presented as mean ± standard deviation. Group 1: patients with chronic obstructive pulmonary disease; Group 2: healthy control group.

Abbreviations: BMI, body mass index; CRP, C‐reactive protein; FEV1, forced expiratory volume in the first second; FVC, forced vital capacity; HCO_3_, bicarbonate level in arterial blood; PCO_2_, partial pressure of carbon dioxide in arterial blood; PO_2_, partial pressure of oxygen in arterial blood; Sat O_2_, oxygen saturation of arterial blood.

**FIGURE 1:**
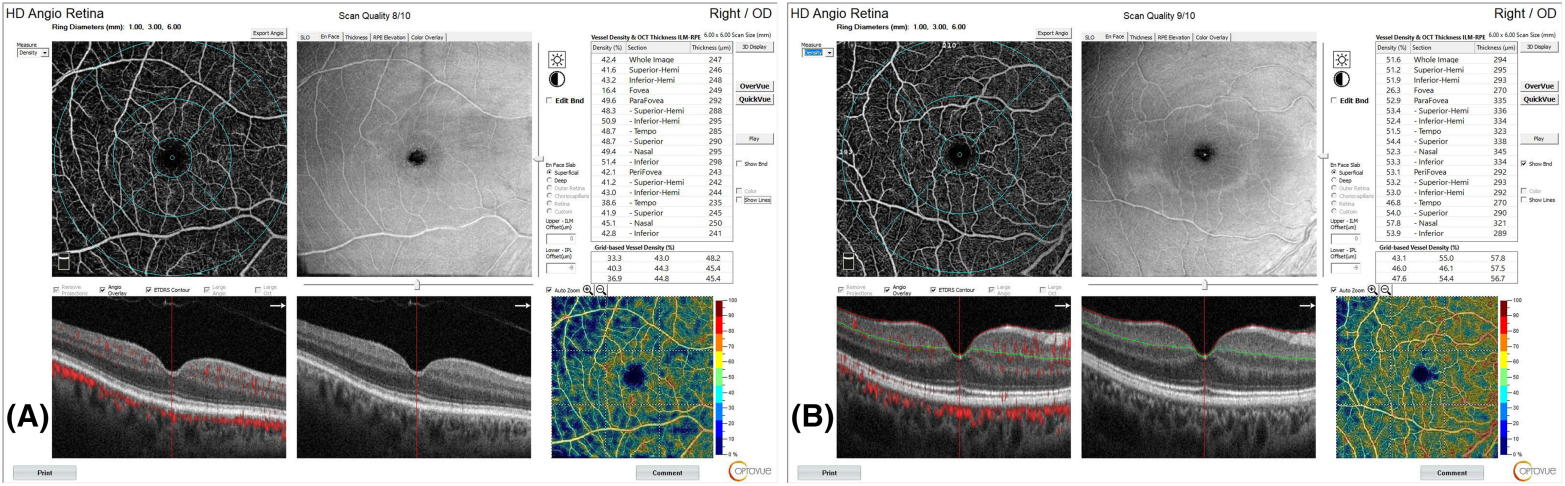
Macular vascular density findings and images of the cases obtained by optical coherence tomography angiography. (A) A patient from the healthy control group. (B) A patient from the chronic obstructive pulmonary disease group

**FIGURE 2:**
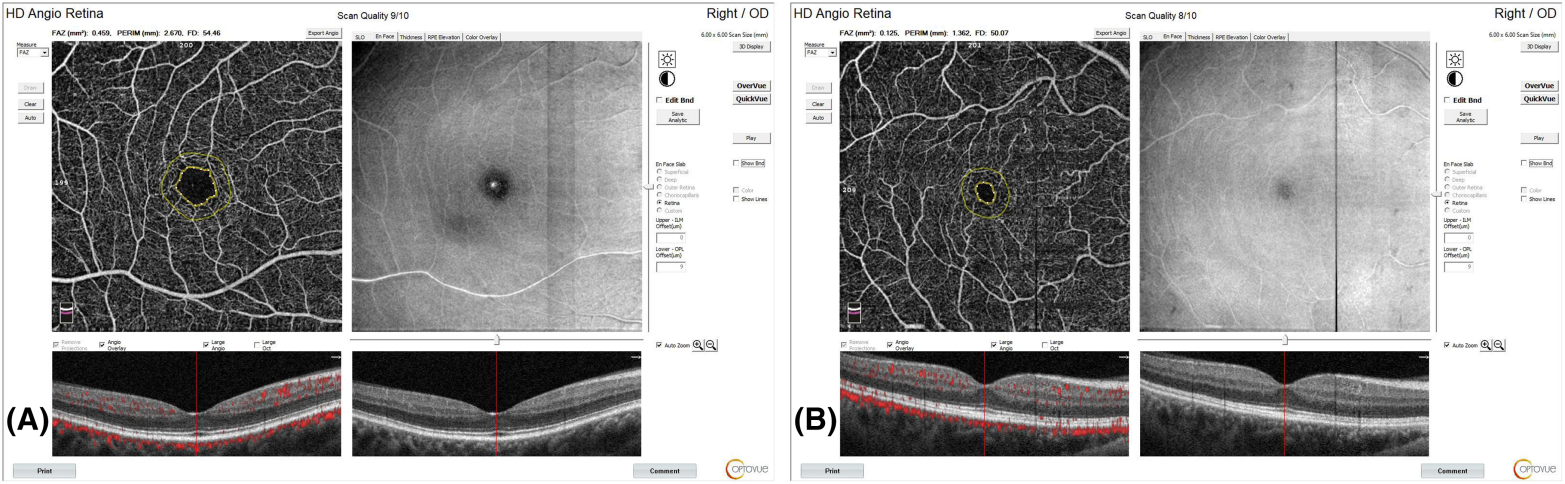
Foveal avascular zone findings and images of the cases obtained by optical coherence tomography angiography. (A) A patient from the healthy control group. (B) A patient from the chronic obstructive pulmonary disease group

The evaluation of SCP and DCP VDs with OCTA showed that there was a significant decrease in all quadrants in the COPD group compared with the control group (Table 2 and Figure 1). Moreover, significant differences were seen in the measurements of FAZ (*p* = 0.002), perimetry (*p* = 0.002), and FD‐300 (*p* = 0.004) between the control and COPD groups (Table 2 and Figure 2).

**TABLE 2:** Superficial, deep, and peripapillary vascular densities, and FAZ and FD‐300 values in optical coherence tomography angiography imaging of the patients

	Vascular density	Group 1 (*n* = 70)	Group 2 (*n* = 71)	*p* value
Superficial capillary plexus values	Whole macula	46.48 ± 4.23	48.81 ± 4.41	0.002
	Inf hemisphere	46.51 ± 4.53	49.07 ± 4.61	0.001
	Sup hemisphere	46.39 ± 4.43	48.56 ± 4.41	0.004
	Fovea	19.33 ± 7.27	22.19 ± 7.08	0.019
	Parafovea	48.82 ± 5.89	51.14 ± 5.07	0.013
	Parafovea temp	48.17 ± 6.51	50.30 ± 5.49	0.037
	Parafovea sup	49.78 ± 6.47	52.02 ± 5.37	0.027
	Parafovea nas	47.74 ± 6.71	50.27 ± 5.98	0.019
	Parafovea inf	47.27 ± 4.13	49.70 ± 4.31	0.015
	Perifovea	47.16 ± 5.33	49.98 ± 4.87	0.001
	Perifovea temp	43.90 ± 5.34	45.64 ± 4.83	0.045
	Perifovea sup	51.23 ± 4.12	53.69 ± 4.38	0.001
	Perifovea nas	51.23 ± 4.12	53.69 ± 4.38	0.001
	Perifovea inf	47.16 ± 5.33	49.98 ± 4.87	0.001
Deep capillary plexus values	Whole macula	46.24 ± 5.84	49.54 ± 6.62	0.002
	Sup hemisphere	45.83 ± 5.96	49.05 ± 6.70	0.003
	Inf hemisphere	46.55 ± 6.46	50.01 ± 6.88	0.003
	Fovea	34.77 ± 7.91	37.61 ± 7.22	0.028
	Parafovea	51.07 ± 5.56	53.76 ± 5.66	0.005
	Parafovea temp	51.46 ± 6.23	54.35 ± 4.79	0.002
	Parafovea sup	50.18 ± 7.31	53.33 ± 6.66	0.008
	Parafovea nas	52.39 ± 6.37	54.60 ± 6.46	0.019
	Parafovea inf	50.30 ± 5.72	52.73 ± 6.88	0.024
	Perifovea	47.54 ± 6.15	51.08 ± 7.08	0.002
	Perifovea temp	50.51 ± 6.00	53.38 ± 6.18	0.006
	Perifovea sup	46.08 ± 6.87	49.98 ± 7.60	0.002
	Perifovea nas	46.15 ± 6.96	49.70 ± 8.02	0.006
	Perifovea inf	47.41 ± 7.64	51.35 ± 7.99	0.003
Peripapillary vascular density values	Whole	48.75 ± 2.96	50.51 ± 2.53	<0.001
	Inside disc	46.76 ± 5.85	49.88 ± 4.73	0.001
	Peripapillary	51.45 ± 3.59	52.95 ± 2.76	0.006
	Sup hemisphere	51.87 ± 3.57	53.49 ± 3.04	0.004
	Inf hemisphere	50.99 ± 4.15	52.65 ± 3.09	0.008
	Nasal sup	48.77 ± 5.35	50.70 ± 3.65	0.016
	Nasal inf	48.01 ± 5.14	48.97 ± 4.70	0.249
	Inferior nasal	51.00 ± 6.47	51.70 ± 5.08	0.473
	Inferior temporal	56.98 ± 4.83	58.12 ± 3.83	0.124
	Temporal inferior	49.88 ± 6.05	53.32 ± 4.04	<0.001
	Temporal superior	54.92 ± 4.45	56.47 ± 3.60	0.025
	Superior temporal	54.19 ± 6.60	56.58 ± 4.41	0.013
	Superior nasal	50.54 ± 6.17	51.47 ± 5.05	0.328
FAZ and FD‐300 values	FAZ	0.28 ± 0.96	0.23 ± 0.84	0.002
	Perimetry	2.09 ± 0.47	1.86 ± 0.40	0.002
	FD‐300	49.37 ± 7.86	51.12 ± 7.33	0.004

*Note*: Continuous data are presented as mean ± standard deviation. Group 1: patients with chronic obstructive pulmonary disease; Group 2: healthy control group.

Abbreviations: FAZ, foveal avascular zone; FD‐300, capillary vessel density covering 300 μm around the fovea.

**FIGURE 3:**
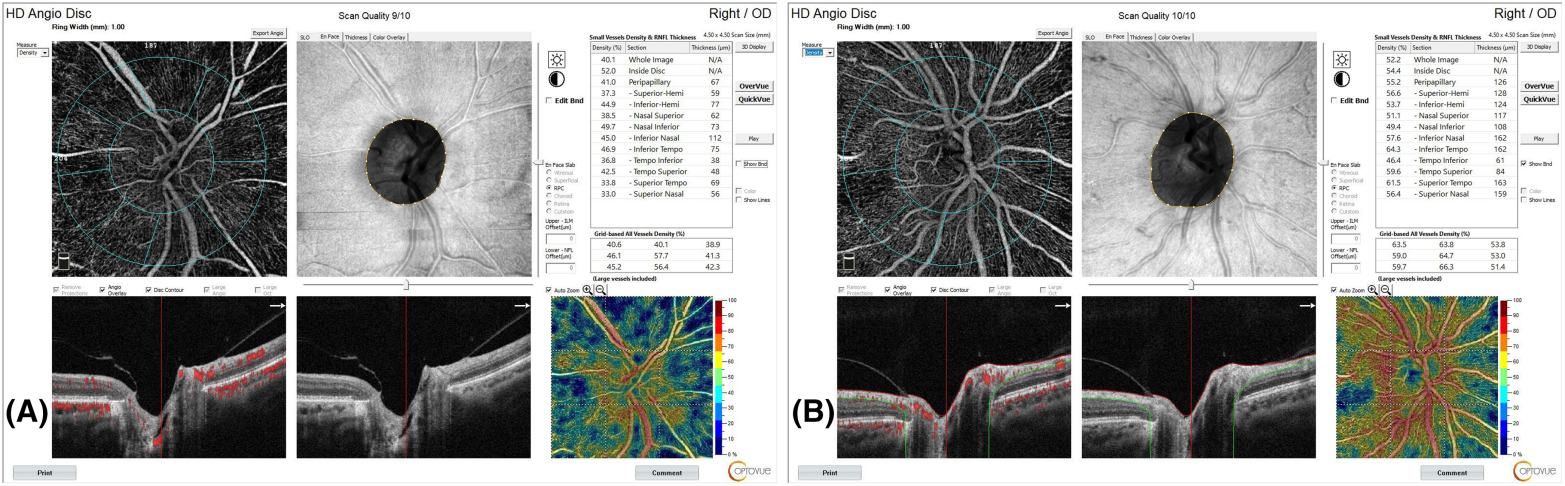
Optical disc radial peripapillary capillary findings and images of the cases obtained by optical coherence tomography angiography. (A) A patient from the healthy control group. (B) A patient from the chronic obstructive pulmonary disease group

There was a significant difference between the control and COPD groups in 4.5 mm angiodisc scans, the whole optic disc (*p* < 0.001), the peripapillary region (*p* = 0.006), and the inside disc (2 mm center of the optic disc; *p* = 0.001). The scans performed in eight quadrants showed a decrease in VD in the COPD group compared with the control group, especially in the temporal region (temporal superior, *p* = 0.025; temporal inferior, *p* < 0.001) (Table 2 and Figure 3).

**FIGURE 4:**
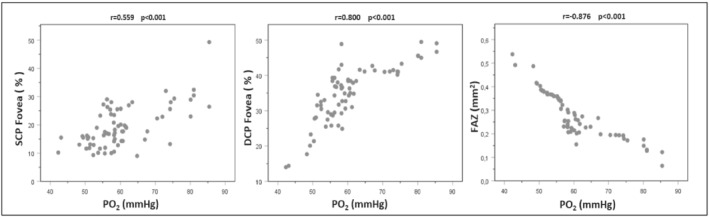
Correlation of PO_2_ values of chronic obstructive pulmonary disease patients with superficial capillary plexus (SCP) foveal vascular density, deep capillary plexus (DCP) foveal vascular density, and foveal avascular zone (FAZ) width

In our study, when we considered the correlation between partial pressure of oxygen in arterial blood (PO_2_) and peripapillary, superficial, and deep VD parameters, we found that VD of the SCP was moderately correlated with the fovea and highly correlated with the parafoveal and perifoveal VD. In DCP VD, a highly positive correlation was found between foveal, parafoveal, and perifoveal VD and peripapillary VD. A highly negative correlation was found between FAZ and PO_2_ (Table 3 and Figure 4).

**TABLE 3:** Correlation of PO_2_ values of COPD patients with OCTA data

Vascular density	PO_2_	
	*r*	*p*
SCP fovea	0.559	<0.001
SCP parafovea	0.772	<0.001
SCP perifovea	0.831	<0.001
DCP fovea	0.800	<0.001
DCP parafovea	0.832	<0.001
DCP perifovea	0.900	<0.001
Peripapillary	0.871	<0.001
FAZ	−0.876	<0.001

*Note*: COPD, chronic obstructive pulmonary disease; DCP, deep capillary plexus; FAZ, foveal avascular zone; OCTA, optical coherence tomography angiography; PO_2_, partial pressure of oxygen in arterial blood; SCP, superior capillary plexus.

## 4 DISCUSSION

In our study, we detected a significant decrease in both SCP and DCP VD measurements in OCTA in the COPD group. Moreover, the COPD group had a significant enlargement in FAZ. In the optical disc scans of the COPD group, there was a significant decrease in VD in the center and peripapillary region of the optic disc, especially in the temporal quadrant.

An abnormal inflammatory response to harmful gases and particles in the respiratory tract and lung parenchyma plays a major role in the pathogenesis of COPD. This inflammatory response causes chronic airway obstruction and other problems by disrupting the repair and defense mechanisms of the lung and causing tissue damage.^8^, ^9^ It is thought that protease–antiprotease and oxidant–antioxidant imbalances play an important role in the pathology of COPD.^9^ This imbalance can be caused by inflammation, together with the effect of oxidant substances in cigarettes and genetic alpha‐1 antitrypsin deficiency.^10^

The biggest risk factor in COPD is smoking, and the mortality rate is higher in COPD associated with smoking.^11^ The pathology occurs when the increase in oxidant substances released from activated macrophages and neutrophils due to the oxidant effect of cigarette smoke cannot be eliminated by antioxidants such as superoxide dismutase, glutathione, and vitamin C in the lungs.^12^, ^13^ As a matter of fact, it was observed that oxidative stress markers such as hydrogen peroxide and 8‐isoprostane increased in sputum examinations and expiratory air in COPD, and these substances increased significantly more during attacks.^8^

In a study by Yanagi et al., they found retinal vessel diameter enlargement in those who smoked 20 or more cigarettes a day compared with those who did not smoke less. In addition, the increase in retinal vein diameter was reversed in those who had quit smoking for 10 years.^14^ This is important because smoking is a significant factor in COPD patients.

Elastin is the basic building material in the alveolar walls, and COPD can be damaged by proteases, especially neutrophil elastase.^10^ Moreover, patients with COPD can have decreased level of antiproteases such as tissue matrix metalloprotease inhibitors, alpha‐1 antitrypsin, and alpha‐2 macroglobulin.^12^ It is thought that these are the main factors in the formation of emphysema in patients with COPD.^8^

Physiopathological changes that occur in COPD are airflow restriction, gas exchange abnormalities, mucus hypersecretion, ciliary dysfunction, pulmonary hypertension, and systemic findings.^8^ These physiopathological changes cause symptoms and signs such as shortness of breath, cough, sputum production, decrease in exercise performance, hypoxemia, hypercapnia, and weight loss.

Chronic hypoxia, oxidative stress, inflammatory cytokines, and smoking are thought to be responsible for the endothelial dysfunction seen in COPD patients.^15^ In healthy endothelial tissues, there is a critical balance between endothelin‐1 (ET‐1), which causes vasoconstriction, and nitric oxide (NO), which causes vasodilation.^16^, ^17^ These two are the most important cytokines in providing ocular blood flow and arterial tonus.^18^, ^19^ ET‐1 levels are low in the retina, choroid, and optic nerve in healthy individuals.^16^, ^17^ It is thought that serum and urinary ET‐1 levels increase in COPD patients, and this may lead to chronic hypoxia.^20^, ^21^ This increase in ET‐1 may also be the reason for the decrease in VD we detected in OCTA.

Chronic hypoxia and hypercapnia in COPD patients may affect choroidal and retinal blood flow. There are color Doppler ultrasonography studies showing that retrobulbar hemodynamics was impaired, retrobulbar blood flow was reduced, and resistance to blood flow was increased in both retinal and choroidal circulation in COPD patients.^22^, ^23^, ^24^ In parallel with these studies, our study's OCTA results indicated a significant decrease in VD values of SCP and DCP, optic disc, and peripapillary region, especially in the temporal side, and a significant widening in FAZ in the COPD group. In our study, there was a positive correlation between oxygen pressure and superficial, deep, and peripapillary VDs in the COPD group and a negative correlation with FAZ, indicating that the data worsened with the increase in hypoxia.

COPD patients with diabetes, hypertension, and other cardiovascular diseases were excluded from the study. Again, although smoking is a major risk factor for COPD, 17.1% of the patients we examined were never smokers, 61.4% had quit smoking, and only 21.5% had a current active smoking history. Therefore, we think that retinal changes seen in our study are related to COPD.

Diabetes, sleep‐related respiratory diseases, lower and upper respiratory tract infections, obstructive sleep apnea syndrome, lung cancer, depression, and myocardial infarction are more common in COPD.^3^ Mechanisms of these diseases in COPD include pulmonary and systemic inflammation, inactivity, polycythemia, hypoxia, hypercapnic acidosis, oxidative stress, and corticosteroid use.^25^

Ozcimen et al. found thinning of peripapillary choroidal thickness in COPD patients, and this may be associated with hypoxia and vascular endothelial dysfunction.^26^ In their review study, Vaes et al. stated that there are structural and functional microvascular changes such as increased retinal venular caliber, lower retinal arterial oxygen saturation, and impaired hemodynamics in the eyes of COPD patients, which they suggested were due to inflammation and hypoxia in COPD.^27^

In their study of 35 COPD and 35 healthy controls, Alkan et al. looked at radial peripapillary capillary (RPC) density and retinal nerve fiber layer (RNFL) thickness.^28^ Similar to our study, they found that the inside disc and peripapillary VDs were significantly lower in the COPD group compared with the control group. Moreover, they found that RNFL was thin in all quadrants, but it was significantly thinner in the nasal superior and inferonasal quadrants. In addition, in COPD patients, as a result of examination of the peripapillary capillary density in eight quadrants, they found thinning in all quadrants, except for superotemporal and temporal superior quadrants. Similarly, in our study, we found that there was thinning in all eight quadrants of the peripapillary region in COPD patients. In the studies conducted by Ozcimen et al.^26^ and Ugurlu et al.,^29^ although subfoveal choroidal thickness was thinner in the COPD group than in the control group, this difference was not significant. Moreover, in Ugurlu et al.'s study, the RNFL was thinner in all four quadrants in patients with COPD, but the difference was significant only in the inferior quadrant. Besides, they found that retinal vein width was larger in patients with COPD, but none of the OCT data correlated with oxygen pressure.^29^ In our study, although OCTA revealed a significant decrease in all quadrants in the SCP and DCP in COPD group and a decrease in VD in the peripapillary region, especially in the temporal side, was also detected. Recent studies indicate that changes in the retinal vein diameter can give an idea about the vascular effect and microcirculation of systemic diseases.^30^, ^31^ In this study, although there was no difference in retinal arterioles between the groups, widening of retinal veins was observed in the COPD group. This situation may cause pulmonary hypertension due to hypoxia and chronic inflammation, and this may lead to a decrease in venous return by increasing intrathoracic pressure. Increased vascular resistance may cause dilatation in peripheral veins due to systolic and diastolic dysfunction in the left ventricle.^32^

In this study, patients with diabetes, dementia, and cardiovascular system diseases such as hypertension were excluded from the study. There was no significant difference in CRP levels between the two groups. COPD patients were recruited when they were in a stable condition. Several limitations of this study should be noted. Our number of samples was limited due to the high prevalence of comorbid diseases in these COPD patients and their exclusion from the study. In addition, only including patients that were in stable period of COPD and having a scan quality threshold of 7 and above for the OCTA images also limited the number of samples. Because this study is one of the first to examine COPD patients with OCTA, further studies are needed for better and comprehensively understanding of this relationship.

Hypoxia and systemic inflammation in COPD can seriously affect eye structures such as the retina and choroid. In our study, OCTA scans revealed significant damage in the macula and optic disc in COPD. For this reason, routine administration of easily applicable and reproducible OCTA together with ophthalmologic examination can be very useful in the follow‐up and treatment of COPD patients.

## CONFLICT OF INTERESTS

The authors declare that they have no conflicts of interest.

## ETHICS STATEMENT

All procedures performed in studies involving human participants were in accordance with the ethical standards of the institutional research committee and with the 1964 Declaration of Helsinki and its later amendments or comparable ethical standards. Informed consent was obtained from all individual participants included in the study.

## AUTHOR CONTRIBUTIONS

M. S. S. and Y. S. İ. contributed to the protocol development and data analysis and drafted the manuscript. M. S. S., Y. S. İ., S. A. B., and H. A. B. contributed to the study conception and participated in its coordination. M. S. S., Y. S. İ., B. Ç., and M. Ç. contributed to the data checking and information retrieval. All the authors designed the study, contributed to the overall management of the study, reviewed the manuscript, and approved the final version of the manuscript.

## Data Availability

The data that support the findings of this study are available on request from the corresponding author. The data are not publicly available due to privacy or ethical restrictions.
